# Discrepancy between antibiotic pack sizes and guideline recommendations: a real-world analysis based on claims data

**DOI:** 10.1007/s15010-024-02420-9

**Published:** 2024-10-23

**Authors:** Sabrina M. Stollberg, Sereina M. Graber, Andreas Kronenberg, Oliver Senn, Stefan Neuner-Jehle, Catherine Pluess-Suard, Carola A. Huber, Andreas Plate

**Affiliations:** 1 Department of Health Sciences, Helsana Group, Zurich, Switzerland; 2 Swiss Centre for Antibiotic Resistance (ANRESIS), Institute for Infectious Diseases, University of Bern, Bern, Switzerland; 3 Institute of Primary Care, University of Zurich and University Hospital Zurich, Pestalozzistrasse 24, Zurich, 8091 Switzerland

**Keywords:** Pack size, Antibiotics, Non-conformity with guidelines, Antibiotic resistance, Healthcare claims data

## Abstract

**Purpose:**

Antibiotics are often only available in predefined pack sizes, which may not align with guideline recommendations. This can result in leftover pills, leading to inappropriate self-medication or waste disposal, which can both foster the development of antibiotic resistance. The magnitude of inappropriate pack sizes is largely unknown. The objective of this study was to evaluate the potential non-conformity of prescribed antibiotic pack sizes.

**Methods:**

This retrospective observational study was based on claims data from a large Swiss health insurance company. The study analysed the prescriptions of eleven different antibiotic substances recommended for the five most common indications for antibiotics in Switzerland. All prescriptions for adult outpatients issued by general practitioners in 2022 were included and extrapolated to the entire Swiss population. Potential non-conformity was defined as a mismatch between the total dosage in a pack and the total dosage recommended.

**Results:**

A total of *n* = 947,439 extrapolated prescriptions were analysed. In 10 of 23 of all analysed substance/indication combinations none of the prescribed packs aligned with the respective guideline recommendation. Considering pack sizes in which the total prescribed dosage of a substance did not correspond to any of the total dosages recommended in at least one of the guidelines, 31.6% of prescriptions were potentially non-conform and an estimated number of 2.7 million tablets were overprescribed.

**Conclusions:**

We found a large discrepancy between prescribed pack sizes and guideline recommendations. Since inadequately prepacked antibiotics may lead to antibiotic resistance and unnecessary waste, efforts are needed to implement alternatives like exact pill dispensing.

**Supplementary Information:**

The online version contains supplementary material available at 10.1007/s15010-024-02420-9.

## Background

The use of antibiotics, whether appropriate or inappropriate, is associated with antibiotic resistance [1], which is considered one of the most urgent problems of our time [2]. It was previously assumed that short-term antibiotic treatment would lead to resistance. However, over the last two decades there has been increasing evidence that the risk of resistance positively correlates with a longer treatment duration. Accordingly, current guidelines specify treatment durations as short as possible [3–10]. In Switzerland, as well as in many other countries, prepacked antibiotics are dispensed instead of exact pill counts. If the number of tablets in a pack exceeds the required amount, it may result in the therapy taking longer than necessary or leaving tablets unused, both of which can pose risks for patients. In particular, left over pills can lead to self-medication and abuse at a later date [11], improper waste disposal to the sewerage, higher costs to society and higher rates of antibiotic resistance [10, 12–14]. In fact, a representative population survey conducted in Switzerland in 2022 revealed that 20% of respondents stated that they would dispose of excess antibiotics in the trash or use them for the next infection [15].

Modelling studies based on theoretical comparisons between on the market available pack sizes and guideline recommendations indicate that non-conform pack sizes are common [12, 16, 17]. An Australian analysis showed, for example, that the available pack sizes could only cover four of 32 guideline recommendations [17]. A Swiss modelling study from 2020 [16] compared pack sizes available in the Swiss pharmaceutical register with Swiss guidelines for antibiotic treatment for the five most frequent infections treated with antibiotics in outpatient care and showed that an adequate antibiotic pack size could be found for only 47% of the considered antibiotic recommendations. A Swiss feasibility report for partial dispensing of antibiotics in times of drug supply shortage stressed the need for further research in this area, especially about the (missing) conformity between guideline recommendations and individual antibiotic pack sizes [18].

However, modelling studies can only analyse what could theoretically have been prescribed and thus, it is unknown to date, either internationally or in Switzerland, which pack sizes are predominantly prescribed for patients in real life. Therefore, the aim of this study was to (1) assess the proportion of prescribed antibiotic pack sizes that were potentially non-conform with guideline recommendations and (2) to determine the corresponding proportion of potential over- and underprescriptions. To address this research question, we aimed to determine the frequency of prescribed antibiotic substances, antibiotic products and pack sizes and compare those to the guideline recommendations of the most frequent indications for antibiotic treatments in Switzerland. Additionally, we aimed to access the frequency of prescribed partial and multiple packs.

## Methods

### Study design and data source

This retrospective observational study is based on healthcare claims data from the Helsana Group as one of the largest health insurance companies in Switzerland covering almost 1.4 million mandatory insured patients from all parts of the country (corresponding to about 15% of the Swiss population).

### Definitions and study population

Antibiotic substance (hereafter referred to as substance) was defined by the World Health Organization (WHO) Anatomical Therapeutic Chemical (ATC) Code of J01 on the fifth level [19]. Antibiotic product (hereafter referred to as product) was specified as a generic with the same substance, dosage per unit (tablets, powder) and number of units. Dosages of units were given in milligram (mg) and total dosages per pack in gram (g). For combinations of substances (e.g., trimethoprim-sulfamethoxazole or combinations of penicillin with β-lactamase inhibitors), the corresponding doses in mg were summed to give one value.

The total dosage per prescribed pack was calculated by multiplying the dosage of one unit by the number of units in a pack. The total dosage recommended in the guidelines was calculated by multiplying the dosage of one unit by the number of recommended doses per day and by the recommended duration of treatment in days. Partial packs were defined as the number of packs (per prescription) not being a whole number (e.g., 0.5 or 1.5 packs). Multiple packs were defined as prescribing more than one pack at a time, taking into account only whole numbers (e.g. 2, 3, 4 packs).

We included antibiotic prescriptions by General Practitioners (GP) that were issued in outpatient settings in 2022, for patients with mandatory health insurance aged 18 years and older. Prescriptions of products coded as non-enteral and non-solid preparations were excluded. When analysing antibiotics recommended exclusively for urinary tract infections (UTI) in women (fosfomycin, nitrofurantoin, norfloxacin), prescriptions for men were excluded from the analyses.

For the main analyses, only prescriptions of single packs were considered. For subanalyses, unless otherwise stated, prescriptions of multiple and partial packs were only included if they did not exceed twice the total amount recommended by any guideline, to exclude prescriptions for chronic treatments.

### Measures and objectives

We selected the most frequent indications for antibiotic treatments in the Swiss general practice [20, 21]: uncomplicated UTI in women (UTI in non-pregnant adult women without functional or anatomical abnormalities of the urogenital system), acute bacterial rhinosinusitis (ABRS), community acquired pneumonia (CAP), streptococcal pharyngitis (SP), and acute otitis media (OM). In a second step, we extracted all first- and second-line treatment recommendations for the five included infections as published in the national guidelines of the Swiss Society of Infectious Diseases as of May 2023 [22]: amoxicillin *(*J01CA04), amoxicillin/clavulanic acid (J01CR02), azithromycin (J01FA10), cefuroxime (J01DC02), clarithromycin (J01FA09), doxycycline (J01AA02), fosfomycin (J01XX01), nitrofurantoin (J01XE01), norfloxacin (J01MA06), penicillin V (J01CE02), and sulfamethoxazole/trimethoprim (J01EE01) (Table 1).

**Table 1:** Treatment recommendations according to Swiss national guidelines (as of May 2023)

	Urinary tract infection^a^	Acute bacterial sinusitis	Community acquired pneumonia	Acute Otitis media	Streptococcal pharyngitis
**First-line recommendations**					
Substance	Nitrofurantoin	Amoxicillin	Amoxicillin	Amoxicillin	Penicillin V
Dosage (mg)	100	1000	1000	1000	625
Times per day	2	2 or 3	3	3	2
Duration (days)	5	5 to 7	5	5	6
Substance	Trimethoprim, Sulfamethoxazole	Amoxicillin, clavulanic acid^b^	Amoxicillin, clavulanic acid		Amoxicillin
Dosage (mg)	960	1000	1000		1000
Times per day	2	2	3		2
Duration (days)	3	5 to 7	5		6
**Second-line recommendations**					
Substance	Fosfomycin	Cefuroxime	Doxycycline	Amoxicillin, clavulanic acid	Cefuroxime
Dosage (mg)	3000	500	100	1000	500
Times per day	1	2	2	3	2
Duration (days)	1	5 to 7	5	5	6
Substance	Norfloxacin	Doxycycline	Azithromycin	Cefuroxime	Clarithromycin
Dosage (mg)	400	100	500	500	500
Times per day	2	2	1	2	2
Duration (days)	3	5 to 7	3	5	6
Substance	Cefuroxime		Clarithromycin	Trimethoprim, Sulfamethoxazole	
Dosage (mg)	500		500	960	
Times per day	2		2	2	
Duration (days)	3		5	5	
Substance	Amoxicillin, clavulanic acid				
Dosage (mg)	625				
Times per day	3				
Duration (days)	3				

^a^Referring to uncomplicated infections in women. For nitrofurantoin, fosfomycin and norfloxacin men were excluded from the analysis. ^b^First line in particularly severe cases and immunocompromised patients, ethmoidal, frontal and sphenoidal sinusitis, patients who do not respond to amoxicillin alone within 72 h

The primary objective of the study was to determine the proportion of prescribed antibiotic pack sizes that were potentially non-conform with guideline recommendations. We determined the proportion of potentially non-conform prescriptions, e.g. potentially underprescription or potentially overprescription, considering each combination of substance and indication separately. A prescribed pack size was defined as potentially non-conform if its total dosage did not correspond to the total dosage recommended for the respective indication in the given guideline. If a treatment interval is recommended in the guidelines (such as for ARBS, where the recommended total dosage is between 10 and 21 g), the entire range was defined as potentially conform.

In an exploratory analysis, we aimed to provide a conservative estimate of the potential overprescription of units. As health claims data do not provide information on the indication, we only considered prescriptions in which the total prescribed dosage did not correspond to any of the total dosages recommended in at least one of the guidelines. The next step was to use the closest recommended dosage of one of the guidelines examined as a reference and to classify the prescription as overprescribed or underprescribed, respectively. For guidelines with interval recommendations, the same procedure was followed, and the lower and upper limits were considered. To obtain an estimate of waste in that respect, we calculated the net number of under/ overprescribed units overall and for each substance.

### Extrapolation of the data

All numbers were extrapolated to the entire Swiss population. In order to minimise socio-demographic bias, the extrapolation procedure was based on a stratification into geographical region (i.e. 26 cantons), year, sex and 16 age classes (total of 26 × 2 × 16 = 832 strati) as used in the Swiss risk equalization statistics [23]. The stratum specific weighting factors used for the extrapolation are given by the ratio between the entire Swiss population (census data) and the number of persons insured with Helsana. A more detailed explanation of the procedure can be found in Neuner-Jehle et al. 2021 [24].

### Statistical analysis

Antibiotic prescription patterns were described using appropriate descriptive statistics. We calculated the number of weighted prescriptions and percentage frequency, relative to the total number of prescriptions, for each substance and the 10 most prescribed products, as well as for the prescriptions of single, multiple, and partial packs. Additionally, we calculated the weighted mean age overall and stratified by sex, and the proportion of women per population who were prescribed at least one of the 11 substances.

All analyses were performed using the statistical programming language R, version 4.3.1 (R Foundation for Statistical Computing, Vienna, Austria) [25].

## Results

### Antibiotic prescriptions

**Fig. 1:**
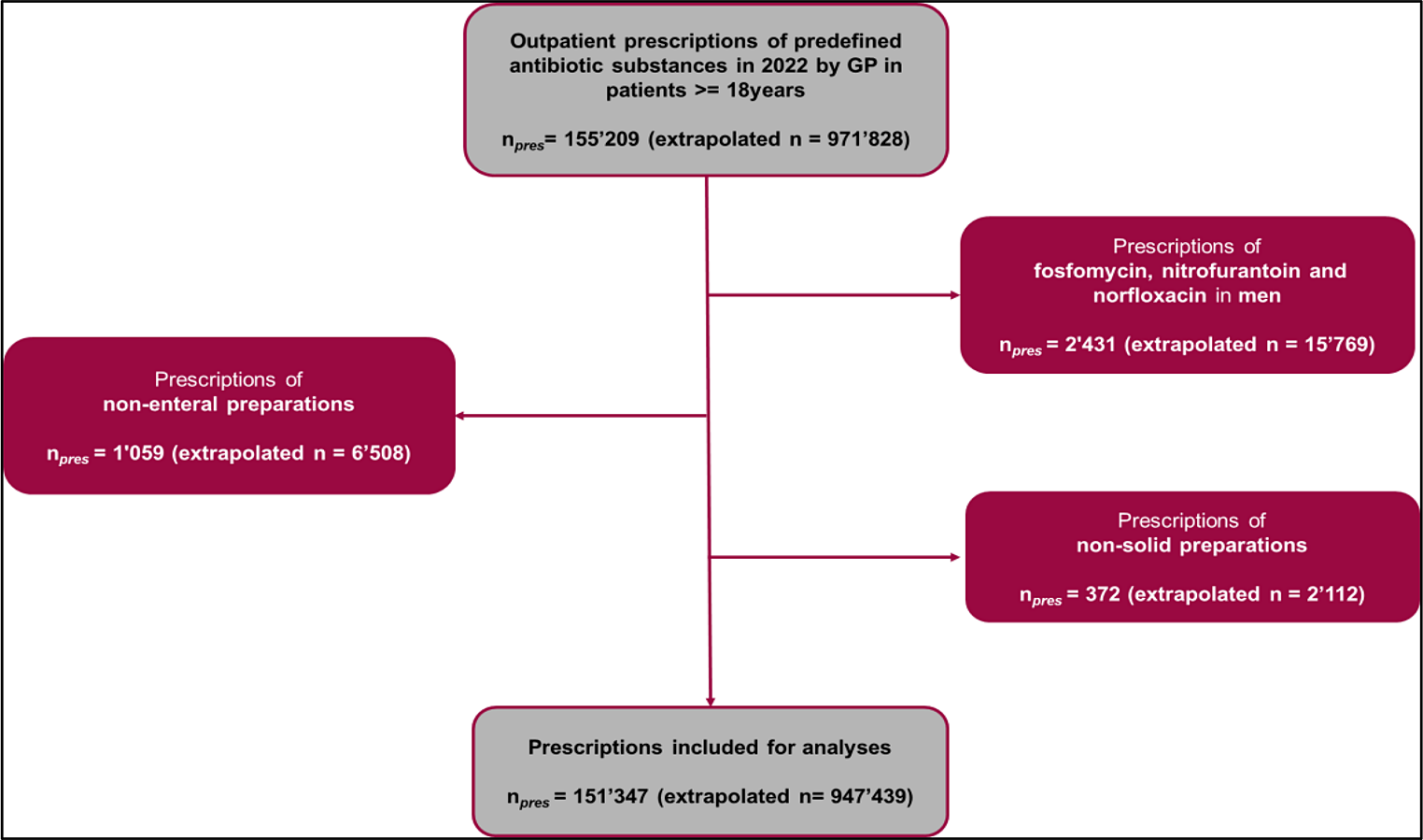
Flow chart in- and exclusion criteria. GP: General practitioner. n_pres_ = Number of prescriptions. Predefined antibiotics substances: Amoxicillin, amoxicillin/clavulanic acid, azithromycin, cefuroxime, clarithromycin, doxycycline, fosfomycin, nitrofurantoin, norfloxacin, penicillin V, and sulfamethoxazole/trimethoprim.

A total of *n* = 151,347 prescriptions from the Helsana database were included, covering *n* = 100,488 patients. The final analysis was based on *n* = 947,439 extrapolated prescriptions, corresponding to *n* = 629,660 patients (Fig. 1). The mean age of antibiotic recipients was 55.4 years, and 64.9% of them were female.

**Fig. 2:**
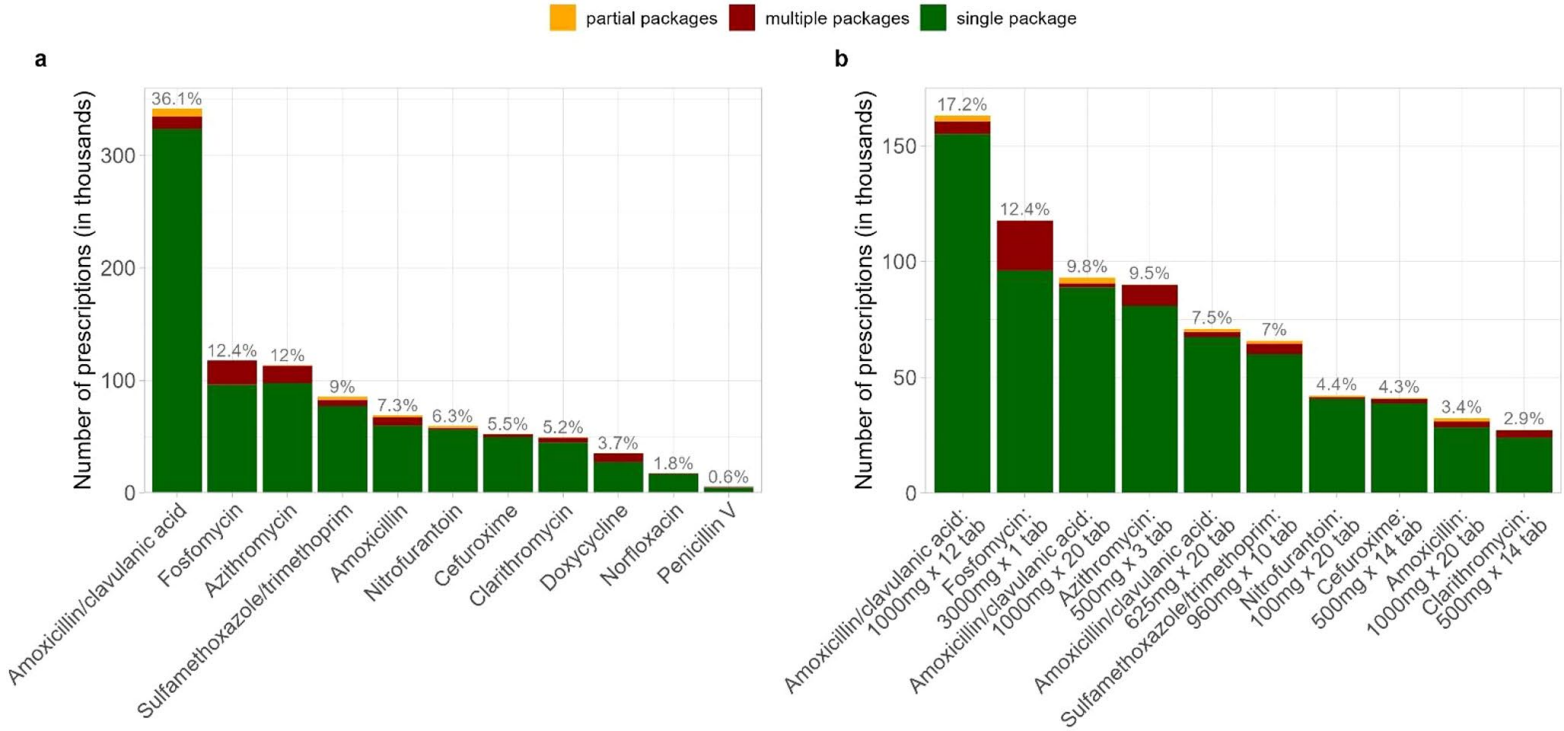
Antibiotic prescription patters by substance (2a) and by product (2b). The percentages indicate the percentage (including single, partial, and multiple pack/s) of all given antibiotic substances (2a) and products (only top 10 ranked are displayed) (2b) prescribed

Antibiotic prescription patterns by substance and product are presented in Fig. 2. Amoxicillin/clavulanic acid (*n* = 341,830 prescriptions, 36.1%) was the most common prescribed substance. Within the substance classes, sulfamethoxazole / trimethoprim had the highest share of partial pack prescriptions (3,082 prescriptions, 3.6%), followed by amoxicillin (2,062, 3.0%) and nitrofurantoin (1,982, 3.3%). In contrast, doxycycline (7,644, 21.6%), fosfomycin (21,593, 18.3%) and penicillin V (921, 17.2%) were the substances with the highest share of multiple pack prescriptions. With exception of doxycycline, norfloxacin and penicillin V, all analysed substances were ranked in the top 10 list of the most common prescribed products (Fig. 2b). For amoxicillin/clavulanic acid, three different products ended up in the top 10 list. Detailed information on prescription patterns as well as on age and sex of antibiotic recipients, stratified by antibiotic substance, are presented in Online Resource 1 and 2.

**Fig. 3:**
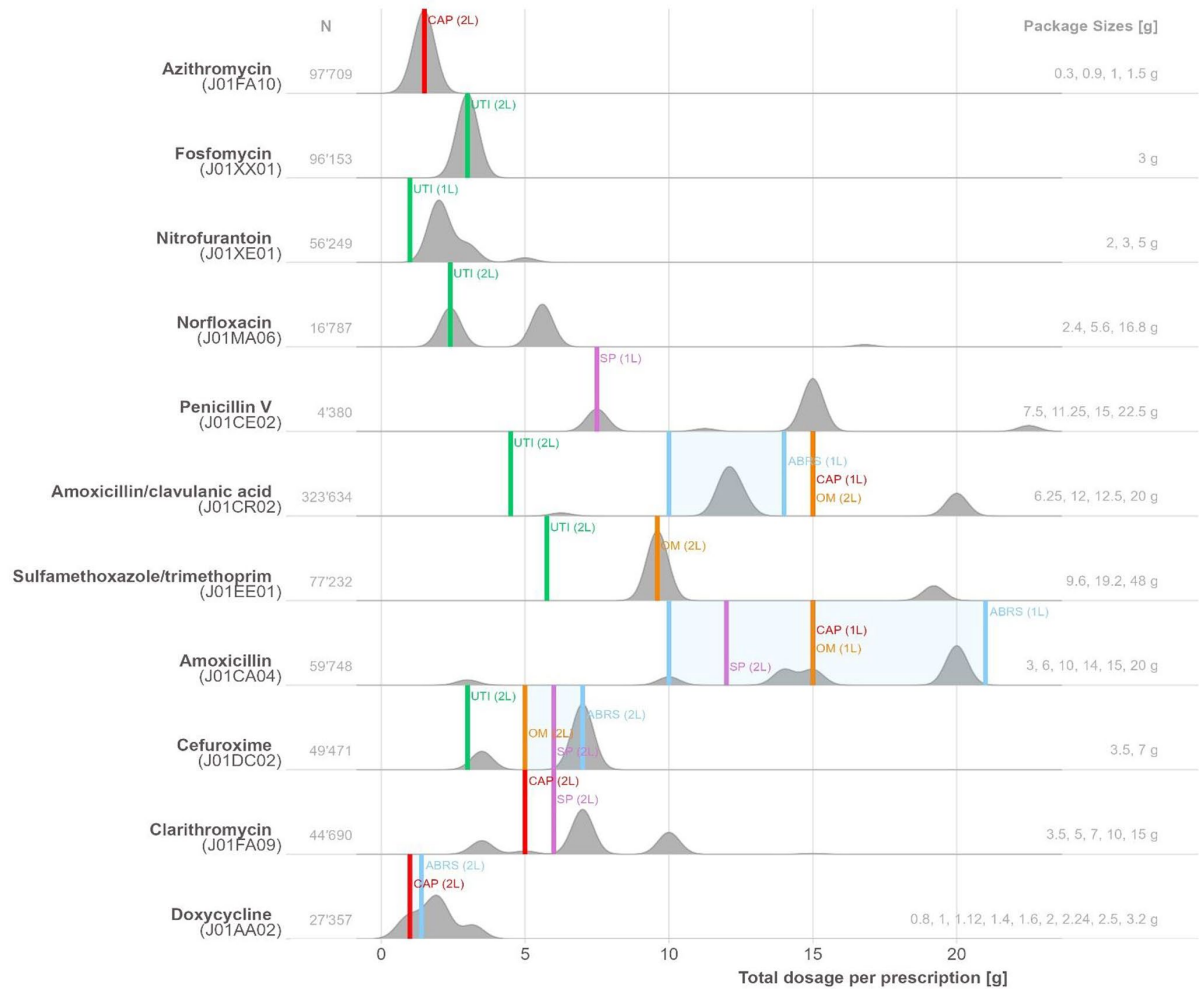
Guideline recommendations versus prescribing practice. Densities of the total dosage per prescription are scaled to 1 so that the maximum point of any density line touches the baseline above. For reasons of readability, numbers on the x-axis are only displayed up to 25 g. In addition, the total number of prescriptions N (left side of the graphic) and the available packs sizes in g (right side) are shown. 1 L: First-line, 2 L: second-line. CAP: community acquired pneumonia, SP: streptococcal pharyngitis, UTI: urinary tract infection, ABRS: acute bacterial rhinosinusitis, OM: Acute otitis media. Reading Example: Azithromycin is a substance prescribed as 2 L treatment for CAP. The vertical red bar corresponds to the total dosage recommended (1.5 g). The grey area represents the density of the total dosage per prescription. The peak of the grey area reaches almost 1 with a steep rise of the curve indicating that almost all prescriptions are conform. In norfloxacin, a 2 L treatment for UTI, only around half of the prescriptions (grey area) is concentrated around the green vertical bar and therefore conform. Another half of the prescriptions is potentially overprescribed and concentrates around 5.6 g. Ranges of treatment recommendations are indicated by the transparent colored areas between two bars, as in ARBS

The proportion of potentially guideline conform prescriptions as well as potentially over- or underprescription differed depending on the substance and the corresponding indication. In 10 out of 23 of the substance-indication combinations examined, none of the prescribed packs met the respective guideline recommendation, resulting in a proportion of potentially conform packs of 0% (Table 2; Fig. 3), e.g. nitrofurantoin for UTI or Amoxicillin for streptococcal pharyngitis. For all other combinations the proportion of conform prescriptions ranged between 3.9% (*n* = 1736 clarithromycin for CAP) and 97.0% (*n* = 94.772, in azithromycin for CAP).

**Table 2:** Potential conformity, over- and under prescribing (non-conformity) by substance and guideline recommendation for the respective indication given are the number of weighted prescriptions and the corresponding percentages representing the proportion of all prescriptions of the given substance. Only prescriptions of single packs were considered

	Urinary tract infection	Acute bacterial sinusitis	Community acquired pneumonia	Acute Otitis media	Streptococcal pharyngitis
First-line recommendations					
Substance	Nitrofurantoin	Amoxicillin	Amoxicillin	Amoxicillin	Penicillin V
Total dosage recommended (g)	1	10 to 21	15	15	7.5
Prescribed pack sizes (g)	2, 3, 5	3, 6, 10, 14, 15, 20	3, 6, 10, 14, 15, 20	3, 6, 10, 14, 15, 20	7.5, 11.25, 15, 22.5
Potentially conform	**0%**	56,055 (93.82%)	11,103 (18.58%)	11,103 (18.58%)	1,151 (26.28%)
Potential underprescription	0%	3,693 (6.18%)	20,284 (33.95%)	20,284 (33.95%)	0%
Potential overprescription	56,250 (100%)	0%	28,361 (47.47%)	28,361 (47.47%)	3,229 (73.72%)
Substance	Trimethoprim, Sulfamethoxazole	Amoxicillin, clavulanic acid	Amoxicillin, clavulanic acid		Amoxicillin
Total dosage recommended (g)	5.76	10 to 14	15		12
Prescribed pack sizes (g)	9.6, 19.2, 48	6.25, 12, 12.5, 20	6.25, 12, 12.5, 20		3, 6, 10, 14, 15, 20
Potentially conform	**0%**	222,558 (68.77%)	**0%**		**0%**
Potential underprescription	0%	12,195 (3.77%)	234,753 (72.53%)		9,356 (15.66%)
Potential overprescription	77,237 (100%)	88,890 (27.47%)	88,890 (27.47%)		50,392 (84.34%)
**Second-line recommendations**					
Substance	Fosfomycin	Cefuroxime	Doxycycline	Amoxicillin, clavulanic acid	
Total dosage recommended (g)	3	5–7	1	15	
Prescribed pack sizes (g)	3	3.5, 7	0.8, 1,1.12,1.4, 1.6, 2, 2.24, 2.5, 3.2	6.25, 12, 12.5, 20	
Potentially conform	96,157 (100%)	38,826 (78.46%)	3,093 (11.31%)	**0%**	
Potential underprescription	0%	10,658 (21.54%)	3,277 (11.98%)	234,753 (72.53%)	
Potential overprescription	0%	0%	20,987 (76.71%)	88,890 (27.47%)	
Substance	Norfloxacin	Doxycycline		Cefuroxime	Cefuroxime
Total dosage recommended (g)	2.4	1-1.4		5	6
Prescribed pack sizes (g)	2.4, 5.6, 16.8	0.8, 1,1.12,1.4, 1.6, 2, 2.24, 2.5, 3.2		3.5, 7	3.5, 7
Potentially conform	7,690 (45.81%)	4,442 (16.24%)		**0%**	**0%**
Potential underprescription	0%	3,277 (11.98%)		10,658 (21.54%)	10,658 (21.54%)
Potential overprescription	9,097 (54.19%)	19,638 (71.78%)		38,826 (78.46%)	38,826 (78.46%)
Substance	Cefuroxime		Azithromycin	Trimethoprim, Sulfamethoxazole	Clarithromycin
Total dosage recommended (g)	3		1.5	9.6	6
Prescribed pack sizes (g)	3.5, 7		0.3, 0.9, 1, 1.5	9.6, 19.2, 48	3.5, 5, 7, 10, 15
Potentially conform	**0%**		94,772 (96.99%)	60,024 (77.71%)	**0%**
Potential underprescription	0%		2,937 (3.01%)	0%	8,701 (19.47%)
Potential overprescription	49,484 (100%)		0%	17,213 (22.29%)	35,989 (80.53%)
Substance (ATC)	Amoxicillin, clavulanic acid		Clarithromycin		
Total dosage recommended (g)	4.5		5		
Prescribed pack sizes (g)	6.25, 12, 12.5, 20		3.5, 5, 7, 10, 15		
Potentially conform	**0%**		1,736 (3.88%)		
Potential underprescription	0%		6,965 (15.59%)		
Potential overprescription	323,643 (100%)		35,989 (80.53%)		

Also: second line

### Explorative analysis

For the explorative analysis we categorized pack sizes as non-conform if the total dosage did not correspond to any of the total dosages recommended in at least one of the guidelines. A weighted mean across all substances resulted in potentially non-conform prescriptions in 31.6% (considering only prescriptions of single packs) or 36.1% (considering in addition prescriptions of partial and multiple packs). Taking partial and multiple prescriptions into account did not change overall appropriateness patterns (Online Resource 3 and 4).

Based on the non-conform prescriptions, we calculated estimates on the overprescribed tablets for each substance (Online Resource 5). For nitrofurantoin, an antibiotic with only a single indication in the outpatient setting, we calculated an estimate of *n* = 780,893 overprescribed tablets. Taking into account all substances, we calculated an estimate of *n* = 2,653,329 overprescribed tablets corresponding to 118,440 overprescribed antibiotic packs.

## Discussion

This retrospective observational study examined the prescribed antibiotic pack sizes and their conformity with the treatment recommendations for the five most common infections treated with antibiotics in Swiss primary care. The study found that in 10 out of 23 of the investigated substance / indication combinations, none of the observed prescriptions aligns with the recommendations in the respective guidelines and that across all substances, 31.6% of all prescriptions were potentially non-conform with any of the guideline recommendations.

To the best of our knowledge, the present study is the first to compare prescribed pack sizes of antibiotics for the most frequent infections for which antibiotics are recommended in a real-world setting based on healthcare claims data. Analysing patterns of prescribed antibiotics in general, we found a high degree of single pack prescriptions, especially for those antibiotics most commonly prescribed in Swiss primary care, e.g. amoxicillin/clavulanic acid. However, unexpectedly, there was a non-negligible level of partial pack prescriptions. In up to 3.6% partial packs were prescribed. This even though there was no legal basis for partial distribution in Switzerland in 2022 and blister packaging or unit dose systems are primarily an option for nursing homes [26]. One might assume that prescribing or dispensing partial packs may be an attempt to circumvent the dilemma of lack of appropriate pack sizes. We are unaware of any national evidence on the behaviour and intentions of physicians on prescribing partial packs of antibiotics. Consequently, this hypothesis should be investigated in further studies. On the other hand, we found a substantial degree of prescribing multiple packs, especially for fosfomycin or doxycycline. The underlying clinical reasons might be diverse, such as prolonged and chronic treatment or the prescription for the travel first aid kit.

We found that for 10 out of 23 of the investigated substance / indication combinations not a single antibiotic prescription was appropriate because no appropriate prepacked pack size is available. The absence of appropriate pack sizes affected both first-line and second-line recommendations. This is of great importance as second-line antibiotics are still frequently prescribed [21, 27]. For instance, in the case of urinary tract infections, which have recently been the most common single indication for the use of antibiotics [21], there are no suitable products available for either of the substances nitrofurantoin and trimethoprim/sulfamethoxazole. Both substances are frequently used [21, 27]. Due to the absence of suitable pack sizes, all patients receive an excessive number of tablets. While leftover nitrofurantoin pills may be used for an additional complete second treatment, the remaining trimethoprim/sulfamethoxazole pills are only enough for two-thirds of a full treatment. However, in both scenarios, there is a risk of inappropriate self-treatment afterwards that in turn may foster AMR development [28]. The same applies to the use of amoxicillin/clavulanic acid in patients with CAP or amoxicillin in patients with SP. Amoxicillin/clavulanic acid is the most commonly used substance for CAP and amoxicillin for SP [21] and for both indications not a single appropriate product is available.

Our results are in line with previous studies, although we found a lower degree of potential non-conform prescriptions [16, 17, 29]. These differences can be explained by the methodologies used. Firstly, in our explorative analysis, we considered all prescriptions with a total dosage in line with any of the treatment recommendations as appropriate, resulting in overall conservative estimates for non-conformity. Secondly, our study relies on real world prescribing data instead of theoretical models and thirdly, different reference guidelines were used. On the other hand, there may be clinical reasons to deviate from treatment recommendations and prescriptions outside the recommendations may be clinically appropriate.

Füri et al. [16] for example, matched available pack sizes with 70 different Swiss guidelines for five common infections in a modelling study. Guidelines were obtained from both national organizations and individual hospitals. They came to the result that for only 47% of guidelines adequate packs were available. A number which is lower than the 57% (13/23 substance indication combinations) of available conform pack sizes in our study and also lower than the weighted mean of potentially conform prescriptions (68.4%) in our explorative analysis. A feasibility study by the Swiss Federal Office of Public Health [18] came to similar results: In the two small, distinct geographic cohorts analysed, adequate pack sizes were available in 65% (*n* = 1,911 prescriptions) and 49% (*n* = 94) of antibiotic dispensations.

### Explorative analysis

Determining the precise number of overprescribed tablets is crucial in understanding the magnitude of the impact of inappropriate pack sizes. However, obtaining this information from routine data is notoriously challenging, as it necessitates knowledge of the prescribed packs, the indication, and ultimately, the doctor’s recommendations to the patient. Theoretically, surplus tablets could be disposed of at pharmacies, reducing their environmental impact and preventing inappropriate use by patients later.

In our exploratory analysis, we estimated the excess tablets for each substance and in sum for all substances. The figures for fosfomycin and nitrofurantoin are likely very close to the true value in the analyzed population of GP, as these substances are almost exclusively used in Switzerland for the analyzed indication. We calculated over 780,000 overprescribed tablets for nitrofurantoin, the most frequently used substance in UTI [21]. As we only analyzed data from GP, it is likely that the number of overprescribed tablets in the entire outpatient setting is much higher, considering that many UTI are also treated in other outpatient settings such as gynecology, emergency departments, walk-in practices and pharmacies.

The estimates of the other substances analyzed may be confounded as they can be used for different indications than those investigated. Especially the substances amoxicillin/clavulanic acid and doxycycline, the two most common substances prescribed in the Swiss outpatient setting, have multiple further indications to use. Nevertheless, these figures provide insight into a problem whose scale cannot yet well be captured by routine data and should be further investigated.

### Implications of the study

The results of this study are in line with previous studies highlighting the fact, that for many indications appropriate pack sizes are lacking. The inadequate pack sizes undermine the numerous antibiotic stewardship interventions aimed at improving the quality of prescribing in outpatient medicine. Countries like the Netherlands [30] or the UK [11] offer the option of dispensing partial packs or a specific number of tablets. In other countries, exact tablet dispensing has recently been evaluated by research teams. For example, in a French cluster randomized trial [31] there was evidence that per unit dispense of antibiotics could not only reduce the number of tablets to reimburse and deposed to the environment but could also improve the treatment adherence of patients. Individual dispensing of antibiotics was also viewed positively by both patients and healthcare providers in a Swiss feasibility study [18]. For example, it was highlighted that patients were more knowledgeable about their treatment and the importance of the correct dosage.

These dispensing options are likely to be more promising in the long term than continuously adjusting pack sizes, especially considering changing guideline recommendations. For example, in 2024, the SSI changed the guideline for the treatment of urinary tract infections with nitrofurantoin from 2 × 100 mg per day to 3 × 100 mg per day, resulting in a lower level of waste but still equivalent to an overprescription of 5/20 tablets in the smallest available pack size. On the other hand, using pack sizes that adhere to guideline recommendations would be an immediate intervention to improve the quality of prescribing.

The short- and long-term costs of exact pill distribution are regularly discussed in health politics, attempting to determine the additional expense of the dispensing practice or pharmacy, (e.g. due to printing out missing package leaflets), compared to savings from leftovers [18, 31, 32], but exact cost-efficiency analysis are lacking to date. But even if higher short-term costs were demonstrated it is important to consider that, similar to other global pressures such as the climate crisis, the true costs of antibiotic resistance are enormous and can hardly be quantified [30, 33–35].

An up-to-date and comprehensive analysis of the discrepancies between pack sizes and patient needs, as carried out in our study, is particularly important in times of rising healthcare costs, medication shortage and increasing demands on sustainability, and ultimately builds an empirical foundation for political decisions. In fact, since March 2023 the partial dispensing of four antibiotics (amoxicillin, amoxicillin/clavulanic acid, cefuroxime and levofloxacin) is for the first time legally possible due to the situation of medication shortage in Switzerland and could pave the way for further part-quantity levies [32].

### Strengths and limitations

This study analyses prescriptions of Swiss primary care. The analysis is based on a large dataset and the methodology used to extrapolate medication prescriptions to the whole population is well established and has been used before [24, 36]. In addition, age and gender distribution of antibiotic recipients in this study were similar to other analyses of antibiotic prescriptions in Swiss primary care [20]. Accordingly, we assume a high degree of external validity. Compared to theoretical modelling approaches [16], the use of real-life data has the advantage that one can observe what was prescribed, rather than what could have been prescribed.

The main limitation of the study is that health insurance data lack information about the specific indication of the prescribed medication, as the data base lacks diagnoses. Accordingly, we were unaware of the specific indication for each antibiotic prescription. In substance/ indication combinations with a potential appropriate pack size, the true proportion of appropriate pack sizes prescribed for the specific indication might be lower than the numbers reported in this study. For substances with further indications outside the analysed ones, e.g. amoxicillin/clavulanic acid or doxycycline, the true proportion of appropriate packs sizes prescribed may be even higher. We have to acknowledge that treatment recommendations may differ to a certain degree and physicians may use regional guidance or international guidelines instead of the national guidelines provided by the Swiss Society of Infectious Diseases. This may also result in a higher degree of appropriate pack sizes. Similarly, it is possible that pregnant women - for whom there are special guidelines which are not considered in the current study - may be treated by general practitioners, potentially causing a slight bias. However, we assume minimal variation, as many pregnant women in Switzerland are treated by their gynaecologist, and thus do not appear in our sample.

A further limitation of the present study, which is based on reimbursement data, is the inability to differentiate between the antibiotics purchased by patients and those actually consumed. Therefore, the actual amount of waste could vary, potentially exceeding the amount estimated in this analysis, as there are indications that a relevant proportion of patients do not adhere to the prescribed treatment duration [37].

## Conclusion

The present study described a remarkable discrepancy between prescribed pack sizes and guideline recommended treatments for the most common antibiotic indications in outpatient care. Policy makers and stakeholders should be aware of the situation, since inadequately prepacked antibiotics may lead to antibiotic resistance and thus increasing health care costs. Efforts by policy makers, the pharmaceutical industry and healthcare providers are needed to implement alternatives like the opportunity for exact pill dispensing or prescribing of partial packs.

## Electronic supplementary material

Below is the link to the electronic supplementary material.

Supplementary Material 1

## Data Availability

The datasets analysed during the current study are not publicly available due to reasons of individual privacy, legal and regulatory affairs, but are available from the corresponding author on reasonable request.
