# Supplementary material for: Discrepancy between antibiotic pack sizes and guideline recommendations: a real-world analysis based on claims data
Source: Infection. 2024 Oct 23;53(3):1029–39. doi: 10.1007/s15010-024-02420-9 (PMC12137524; doi:10.1007/s15010-024-02420-9)
Supplement: Supplementary file 1 — Supplementary Material 1 [file 15010_2024_2420_MOESM1_ESM.docx]

**Discrepancy between antibiotic pack sizes and guideline recommendations: a real-world analysis based on claims data.**

**Infection.**

Sabrina M. Stollberg, Sereina M. Graber, Andreas O. Kronenberg, Oliver Senn^,^ Stefan Neuner-Jehle, Catherine Pluess-Suard, Carola A. Huber, Andreas Plate

**Corresponding author:**

Andreas Plate, MD

University Hospital Zurich

Institute of Primary Care

Pestalozzistrasse 24

8091 Zurich, Switzerland

[Andreas.Plate@usz.ch](mailto:Andreas.Plate@usz.ch)

**Online Resource**

**Online Resource 1: Detailed prescription patterns of all 11 analyzed antibiotic substances**

Given are the numbers of weighted prescriptions and the corresponding percentages, as well as the weighted mean of age overall, and stratified by sex.

| Substance (ATC) | Prescriptions | Single packs | Partial packs | Multiple packs | Multiple and partial packs ^a^ | Number of products | Mean age | Percentage women |
| --- | --- | --- | --- | --- | --- | --- | --- | --- |
| Amoxicillin/clavulanic acid (J01CR02) | 341,830 (36.1%) | 323,634 (94.7%) | 7,184 (2.1%) | 10,999 (3.2%) | 2,159 (0.6%) | 4 | 54.7 | 54.9% (148,437) |
| Fosfomycin (J01XX01) | 117,764 (12.4%) | 96,153 (81.6%) | 14 (<0.1%) | 21,593 (18.3%) | 3,557 (3%) | 1 | 59.9 | 100% (88,775) |
| Azithromycin (J01FA10) | 113,601 (12%) | 97,709 (86%) | 733 (0.6%) | 15,158 (13.4%) | 3,928 (3.5%) | 5 | 50.4 | 58.4% (53,936) |
| Sulfamethoxazole/trimethoprim (J01EE01) | 85,567 (9%) | 77,232 (90.3%) | 3,082 (3.6%) | 5,248 (6.1%) | 1,120 (1.3%) | 3 | 64.4 | 75.4% (48,656) |
| Amoxicillin (J01CA04) | 69,092 (7.3%) | 59,748 (86.5%) | 2,062 (3%) | 7,282 (10.5%) | 1,005 (1.5%) | 8 | 49.7 | 58.5% (34,653) |
| Nitrofurantoin (J01XE01) | 59,513 (6.3%) | 56,249 (94.5%) | 1,982 (3.3%) | 1,282 (2.2%) | 1,282 (2.2%) | 3 | 64.8 | 100% (46,116) |
| Cefuroxime (J01DC02) | 52,399 (5.5%) | 49,471 (94.4%) | 534 (1%) | 2,382 (4.6%) | 116 (0.2%) | 2 | 53.9 | 64.2% (28,611) |
| Clarithromycin (J01FA09) | 49,422 (5.2%) | 44,690 (90.4%) | 548 (1.1%) | 4,184 (8.5%) | 3,784 (7.7%) | 5 | 51.8 | 58.9% (25,548) |
| Doxycycline (J01AA02) | 35,427 (3.7%) | 27,357 (77.2%) | 425 (1.2%) | 7,644 (21.6%) | 5,217 (14.7%) | 10 | 54.7 | 50.8% (13,456) |
| Norfloxacin (J01MA06) | 17,463 (1.8%) | 16,787 (96.1%) | 139 (0.8%) | 537 (3.1%) | 203 (1.2%) | 3 | 63.5 | 100% (13,773) |
| Penicillin V (J01CE02) | 5,360 (0.6%) | 4,380 (81.7%) | 59 (1.1%) | 921 (17.2%) | 564 (10.5%) | 4 | 37.6 | 64.2% (2,370) |

^a^ Including more than twice the recommended dosage.

**Online Resource 2: Density plots depicting the distributions of prescriptions of partial and multiple packs by substance**

Prescriptions for single packs are excluded. Prescriptions exceeding the double recommended maximum dosage are included.


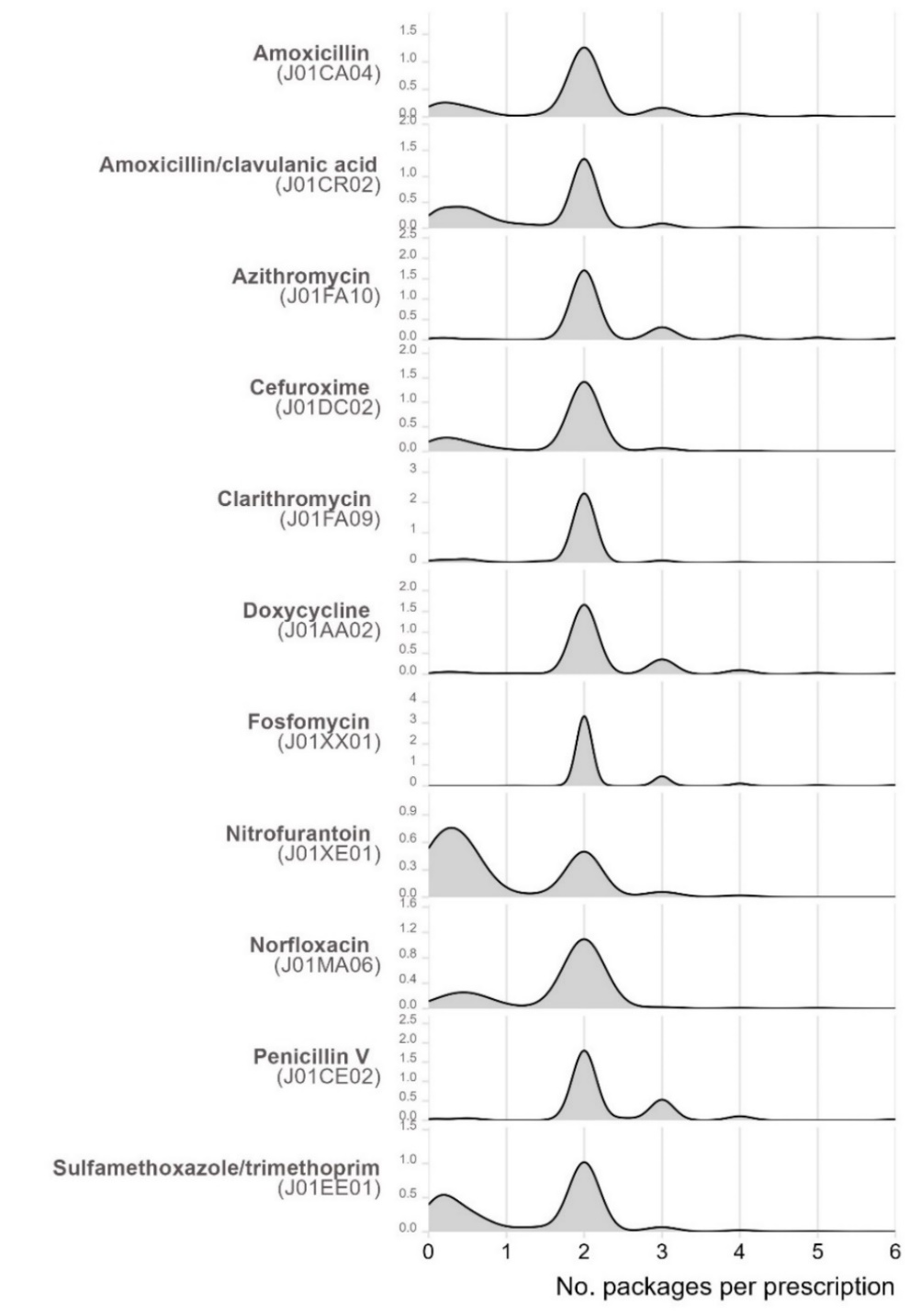


**Online Resource 3: Guideline recommendations versus prescribing practice: Prescriptions of partial and multiple packs included**

Densities are based on weighted numbers of prescriptions and are scaled to 1 so that the maximum point of any density line touches the baseline above. For reasons of readability, numbers on the x-axis are only displayed up to 25g. In addition, the total number of prescriptions N (left side of the graphic) and the available packs sizes in g (right side) are shown. 1L: First-line, 2L: second-line. CAP: community acquired pneumonia, SP: streptococcal pharyngitis, UTI: urinary tract infection, ABRS: acute bacterial rhinosinusitis, OM: Acute otitis media.

Reading Example: Azithromycin is a substance prescribed as 2L treatment for CAP. The vertical red bar corresponds to the total dosage recommended (1.5g). The grey area represents the density of the total dosage per prescription. The peak of the grey area reaches almost 1 with a steep rise of the curve indicating that almost all prescriptions are conform except for a small portion of overprescriptions. In norfloxacin, a 2L treatment for UTI, only around half of the prescriptions (grey area) is concentrated around the green vertical bar and therefore conform. Another half of the prescriptions is potentially overprescribed and concentrates around 5.6g. Ranges of treatment recommendations are indicated by the transparent colored areas between two bars, as in ARBS.


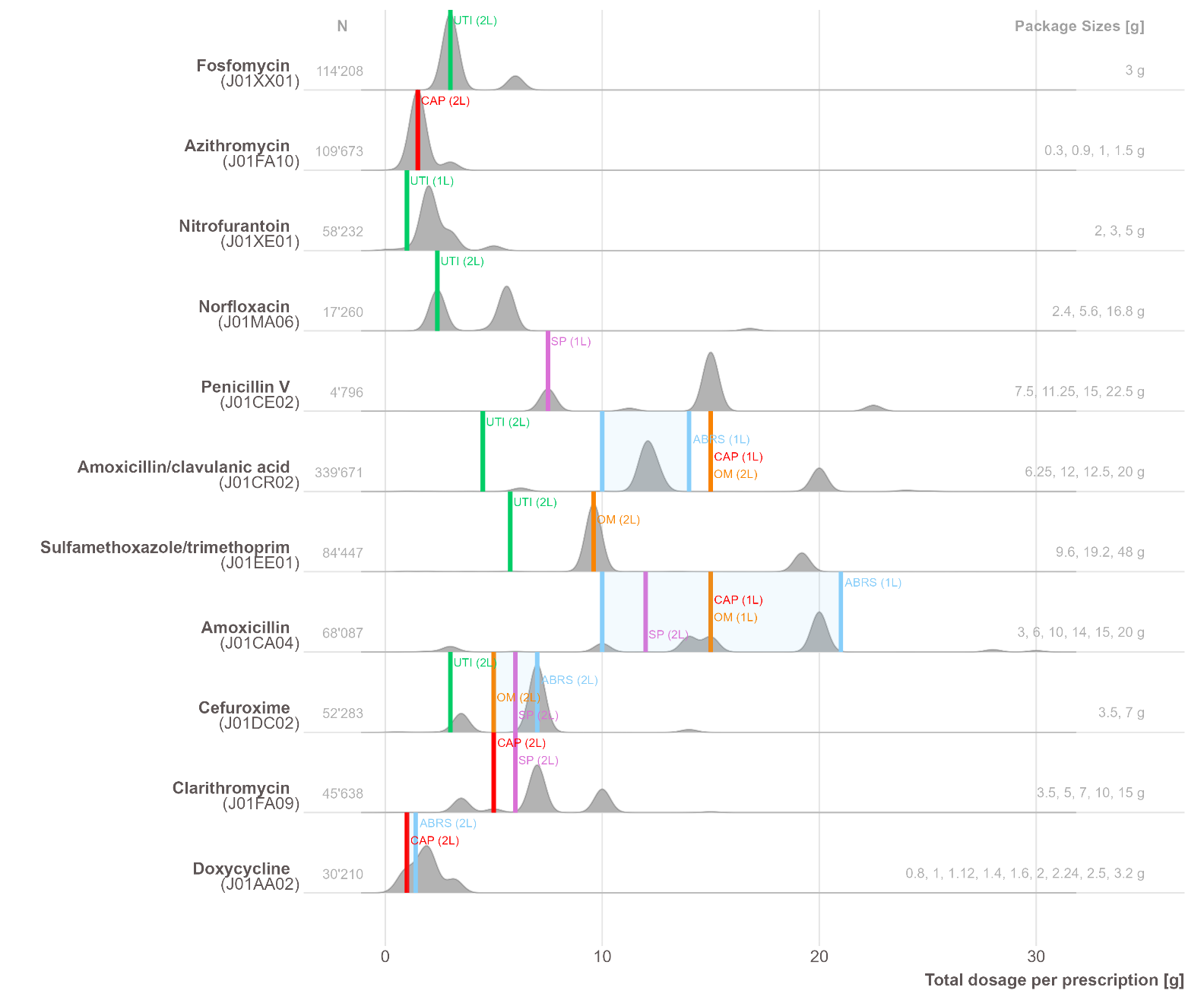


**Online Resource 4: Explorative Analysis: Potential conformity and non-conformity (over- and underprescribing) by substance**

Given are the numbers of prescriptions and the corresponding percentage representing the proportion of all prescriptions in each dataset.

| Substance (ATC) | Inclusion criteria for prescriptions | Conform prescriptions | Non-conform prescriptions | |
| --- | --- | --- | --- | --- |
|  |  |  | Overprescriptions | Underprescriptions |
| Amoxicillin (J01CA04) | all prescriptions^a^ | 57,871 (85%) | 4,895 (7.2%) | 5,320 (7.8%) |
|  | *single* packages only | 56,056 (93.8%) | 0 (0%) | 3,693 (6.2%) |
| Amoxicillin/clavulanic acid (J01CR02) | all prescriptions^a^ | 225,930 (66.5%) | 110,093 (32.4%) | 3,648 (1.1%) |
|  | *single* packages only | 222,558 (68.8%) | 101,084 (31.2%) | 0 (0%) |
| Azithromycin (J01FA10) | all prescriptions^a^ | 94,781 (86.4%) | 11,302 (10.3%) | 3,591 (3.3%) |
|  | *single* packages only | 94,772 (97%) | 0 (0%) | 2,937 (3%) |
| Cefuroxime (J01DC02) | all prescriptions^a^ | 39,399 (75.2%) | 12,504 (23.9%) | 380 (0.7%) |
|  | *single* packages only | 38,826 (78.5%) | 10,658 (21.5%) | 0 (0%) |
| Clarithromycin (J01FA09) | all prescriptions^a^ | 1,854 (4.1%) | 36,521 (80%) | 7,264 (15.9%) |
|  | *single* packages only | 1,736 (3.9%) | 35,989 (80.5%) | 6,965 (15.6%) |
| Doxycycline (J01AA02) | all prescriptions^a^ | 4,555 (15.1%) | 22,159 (73.4%) | 3,496 (11.6%) |
|  | *single* packages only | 4,442 (16.2%) | 19,638 (71.8%) | 3,277 (12%) |
| Fosfomycin (J01XX01) | all prescriptions^a^ | 96,157 (84.2%) | 18,050 (15.8%) | 0 (0%) |
|  | *single* packages only | 96,157 (100%) | 0 (0%) | 0 (0%) |
| Nitrofurantoin (J01XE01) | all prescriptions^a^ | 988 (1.7%) | 56,691 (97.4%) | 553 (0.9%) |
|  | *single* packages only | 0 (0%) | 56,249 (100%) | 0 (0%) |
| Norfloxacin (J01MA06) | all prescriptions^a^ | 7,696 (44.6%) | 9,550 (55.3%) | 14 (0.1%) |
|  | *single* packages only | 7,690 (45.8%) | 9,097 (54.2%) | 0 (0%) |
| Penicillin V (J01CE02) | all prescriptions^a^ | 1,165 (24.3%) | 3,615 (75.4%) | 17 (0.4%) |
|  | *single* packages only | 1,151 (26.3%) | 3,229 (73.7%) | 0 (0%) |
| Sulfamethoxazole/trimethoprim (J01EE01) | all prescriptions^a^ | 60,419 (71.5%) | 23,593 (26.8%) | 1,435 (1.7%) |
|  | *single* packages only | 60,024 (77.7%) | 17,213 (22.3%) | 0 (0%) |

^a^ Single, partial, and multiple packs

**Online Resource 5: Estimates of potentially under- and overprescribed tablets and packs by substance (net number)**

The analyses excluded prescriptions of packs with unit (tablet, powder) dosages that did not match any of the unit dosages recommended in guidelines.

| Substance (ATC code) | Dose (g) | Mean no. of units per package | Total no. of under/ overprescribed units | Total no. of under/ overprescribed packs |
| --- | --- | --- | --- | --- |
| Substances with single indication | | | | |
| Fosfomycin (J01XX01) | 3 | 1 | 0 | 0 |
| Azithromycin (J01FA10) | 0.5 | 3 | 0 | 0 |
| Nitrofurantoin (J01XE01) | 0.1 | 20 | 780,893 | 26,030 |
| Norfloxacin (J01MA06) | 0.4 | 6 | 86,277 | 3,081 |
| Penicillin V (J01CE02) | 0.625 | 18 | 33,455 | 1,394 |
| Substances with multiple indications | | | | |
| Amoxicillin (J01CA04) | 1 | 6.5 | -18,195^*^ | -6,065^*^ |
| Amoxicillin/clavulanic acid (J01CR02) | 0.625 | 10 | 753,428 | 50,229 |
| Amoxicillin/clavulanic acid (J01CR02) | 1 | 12 | 444,448 | 22,222 |
| Cefuroxime (J01DC02) | 0.5 | 14 | 0 | 0 |
| Clarithromycin (J01FA09) | 0.5 | 17 | 148,729 | 7,436 |
| Doxycycline (J01AA02) | 0.1 | 9 | 125,386 | 5,573 |
| Sulfamethoxazole/trimethoprim (J01EE01) | 0.96 | 15 | 298,908 | 8,540 |
| Total | | | 2,653,329 | 118,440 |
|  | | |  |  |

*: Amoxicillin: prescriptions of single packs only show underprescriptions relative to the closest guideline.

0: all prescriptions of exactly 1 pack correspond to a guideline, therefore no units are under/overprescribed.
